# Noise-induced vestibular dysfunction in rats: longitudinal assessment using cVEMP and behavioral testing after low-frequency acoustic trauma

**DOI:** 10.3389/fnint.2025.1677019

**Published:** 2026-01-05

**Authors:** Fatma Nur Komur, Bugra Genc, Luis Roberto Cassinotti, Gabriel Corfas, Ayca Ciprut, Ali Cemal Yumusakhuylu

**Affiliations:** 1 Department of Audiology and Speech Disorders, Institute of Health Sciences, Marmara University, Istanbul, Türkiye; 2 Department of Otolaryngology-Head and Neck Surgery, Kresge Hearing Research Institute, University of Michigan, Ann Arbor, MI, United States; 3 Department of Laboratory Animals, Faculty of Veterinary Medicine, Ondokuz Mayis University, Samsun, Türkiye; 4 Department of Audiology, Faculty of Medicine, Marmara University, Istanbul, Türkiye; 5 Department of Otolaryngology, Faculty of Medicine, Marmara University, Istanbul, Türkiye

**Keywords:** noise-induced trauma, vestibular evoked potentials, vestibular dysfunction, cVEMP, behavioral assessment

## Abstract

**Background and objective:**

High-intensity noise exposure is a well-established risk factor for auditory dysfunction; however, its effects on the vestibular system remain poorly understood. This is an important question due to the anatomical proximity and shared vulnerability of cochlear and vestibular structures. This study aims to determine the longitudinal effects of prolonged low-frequency noise (LFN) exposure at two different sound intensities (110- and 120-dB SPL) on vestibular function in Sprague-Dawley rats using behavioral and electrophysiological assessments.

**Materials and methods:**

Adult male Sprague-Dawley rats (3-months-old) were exposed to LFN (0.5–4.0 kHz) at either 110- or 120-dB SPL for 6 h and monitored over 21 days. Cervical vestibular-evoked myogenic potentials (cVEMPs), auditory brainstem responses (ABRs), and balance-related behaviors were evaluated at baseline and different times after exposure.

**Results:**

Exposure to 120 dB SPL resulted in significant and permanent vestibular dysfunction, evidenced by elevated cVEMP thresholds and reduced cVEMP P1-N1 suprathreshold amplitudes. These parameters partially recovered over 21 days but did not return to baseline levels. As expected for this noise exposure, large ABR thresholds increases and peak I amplitudes reductions were observed. In addition, behavioral tests showed impaired motor coordination over 21 days. In contrast, 110 dB SPL exposure only caused temporary cVEMP P1-N1 amplitude decreases and much smaller ABR threshold increases.

**Conclusion:**

These results show that, similar to the auditory system, LFN exposure has an intensity-dependent effect on vestibular function and highlight the importance of including vestibular evaluations for a comprehensive assessment of noise-induced health conditions.

## 1 Introduction

High-intensity noise exposure is a significant public health concern associated with various medical conditions (Basner et al., 2014). While most attention has been focused on the effects of noise on hearing (Themann and Masterson, 2019), recent studies suggest that noise may also disrupt vestibular structures and function, likely due to the anatomical and physiological proximity of the cochlear and vestibular organs (Golz et al., 2001; Zhu et al., 2011).

Otolith organs, the saccule and utricle, have been shown to be sensitive to acoustic stimulation, including the vestibulo-collic reflex pathway (Curthoys et al., 2011; McCue and Guinan, 1995). Recent clinical studies reported that noise-exposed individuals, with or without permanent hearing loss, can exhibit vestibular dysfunction as assessed by cervical vestibular myogenic evoked potentials (cVEMP) (Akin et al., 2012; Kumar et al., 2010; Macena Duarte et al., 2022; Snapp et al., 2023; Tseng and Young, 2013; Wu and Young, 2009). Animal studies revealed that intense noise exposure can cause damage to vestibular structures (Akdogan et al., 2009; Hsu et al., 2008; Lau and Vasconcelos, 2023; Stewart et al., 2016, 2020a,b; Tamura et al., 2012).

Low-frequency noise (LFN) is a common acoustic stressor generated in both environmental and occupational settings, including transportation systems, industrial machinery, household appliances, and building services (Berglund et al., 1996). LFN represents a challenge in the context of industrial noise control and environmental health, due to its long wavelength, strong resonance properties, and lower absorption by barriers (Findeis and Peters, 2004; Leventhall et al., 2003).

Current evidence suggests that exposure to LFN can have adverse effects not only on the auditory system but also on cardiovascular and neurological function (Araújo Alves et al., 2020). Animal studies have reported that LFN exposure can cause damage to otolith organs, e.g., hair cell stereocilia loss and reduction in calyx afferent terminals (Stewart et al., 2016, 2020a). These alterations are associated with transient impairments in vestibular-evoked potentials and functional deficits. Therefore, understanding the long-term effect of LFN on vestibular health is essential for developing effective protective strategies.

Animal studies examining the effects of noise on vestibular function have predominantly focused on vestibular short-latency evoked potentials (VsEPs) (Stewart et al., 2016, 2018, 2020a,^2021^). VsEPs are considered a reliable electrophysiological measure generated by frequently repeated head jerk stimuli at varying intensities (Jones et al., 1999; Mock et al., 2011). However, the clinical applicability of these responses to routine vestibular assessments in humans has not yet been established. Therefore, it is important to focus on reliable measurement of cVEMPs in rodent models, given their ease of measurement and common use in clinical practice. In the present study, we focus on the long-term effects of noise exposure by measuring cVEMP responses with specialized test techniques. Furthermore, we examine the potential associations between auditory function and balance-related behaviors in a longitudinal framework.

## 2 Materials and methods

### 2.1 Experimental animals

All experimental procedures were conducted on 12-weeks-old male Sprague-Dawley rats (400–600 g) maintained on a 12:12-h light-dark cycle with unrestricted access to food and water. The ambient noise level was maintained below 70 dB SPL (quiet room), with the temperature kept between 20 °C and 26 °C and relative humidity around 45%–65%. All protocols adhered to NIH guidelines and received approval (49.2023mar) from the Marmara University Institutional Animal Care and Use Committee.

### 2.2 Noise calibration and exposure

The acoustic stimulus was adapted to target low-frequency bands (0.5–4.0 kHz, spanning three octaves), corresponding to the most apical 20% of the rat cochlea and the lower edge of their hearing range, following established methodologies (Bartikofsky et al., 2023; Stewart et al., 2016, 2020a). Noise was generated using Audacity (v3.5.1), and its spectral content and intensity were verified and adjusted using a Bruel & Kjaer sound level meter (Type 2235) equipped with a microphone (Type 4176) and an octave filter set (Type 1624), with measurements taken at various cage locations. Following calibration, the noise reached its expected characteristics, exhibiting a sound pressure level of either 110 or 120 dB across the 0.5–4 kHz frequency range.

For noise exposure, unanesthetized rats were housed in pairs in separate wire mesh cages, arranged to receive noise from all directions. The cages were placed approximately 20 cm from a loudspeaker (KL10 series, dBTechnologies, Italy) driven by a StageArt Audio SMR6 amplifier connected to a computer audio source. Noise exposure was carried out in a custom-built exposure chamber (1 × 1 × 1 m) that was double-walled, soundproof, anechoic, and equipped with adequate ventilation. During the noise exposure, animals were regularly monitored through a translucent window.

### 2.3 Experimental design

**FIGURE 1:**
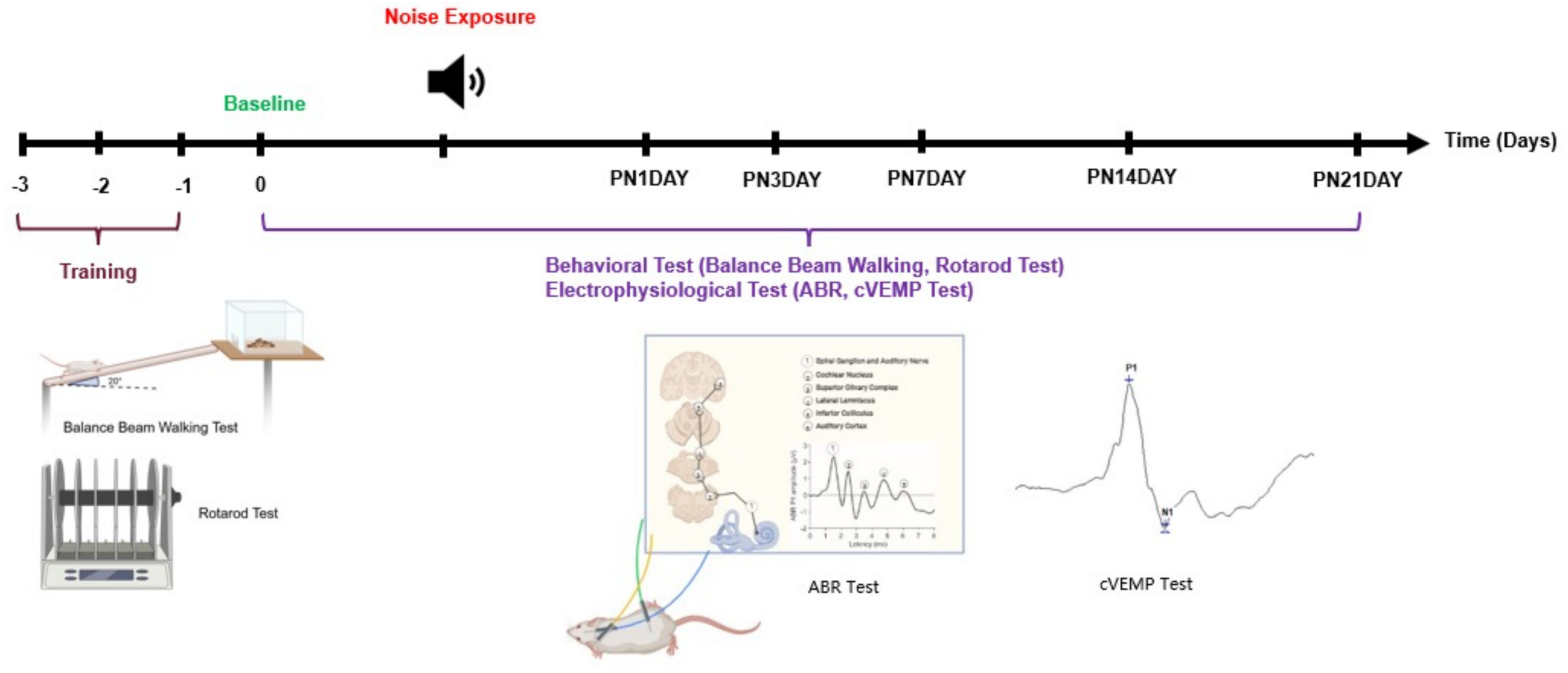
Experimental timeline and protocol for behavioral and electrophysiological assessments. All animals underwent behavioral training, including balance beam walking and rotarod tasks, for three consecutive days prior to noise exposure (days –3 to –1). Baseline assessments were performed on day 0. On the following day, animals were subjected to noise exposure. Post-exposure, the animals were monitored and evaluated over a 21-days period (PN1 to PN21). During this period, behavioral (balance beam walking, rotarod test) and electrophysiological evaluations (ABR and cVEMP) were conducted at multiple time points to assess functional changes. Created in BioRender. Kömür (2025) https://BioRender.com/oa8b6o9.

To evaluate vestibular function before and after noise exposure, behavioral and electrophysiological assessments were conducted at six timepoints: pre-noise baseline (Day 0), and sequentially on the first (PN1DAY), third (PN3DAY), seventh (PN7DAY), fourteenth (PN14DAY), and twenty-first day post noise (PN21DAY) (Figure 1). All animals were trained 3 days before baseline testing to ensure consistent task performance. After training, the animals were randomly divided into three groups. Group 1 (*n* = 7) was exposed to a continuous 120 dB SPL LFN for 6 h. Group 2 (*n* = 6) was exposed to continuous 110 dB SPL LFN, Group 3 (control-sham) (*n* = 6) was kept in the sound booth for 6 h without noise exposure. These animals were included in the behavioral analyses to control potential influences of the experimental environment, stress, and time-related fatigue, as well as for natural variations that may occur in behavior, thereby allowing direct comparison with the noise-exposed groups. The following sections describe the protocols and tests used in the experimental process.

### 2.4 Behavioral measurements

#### 2.4.1 Balance beam walking test

The beam walking test evaluated the animals’ motor coordination and balance skills. The beam had a diameter of 25 mm and a length of 110 cm, located at 75 cm from the ground (Manno et al., 2023). To encourage the motivation of the beam crossing, the animal’s home cage was used as the target point. To increase the difficulty level of the test, the beam was placed at an angle of 20° from the platform. Three separate trials were recorded, and the average of the obtained data was used in the analysis. The beam crossing time from the beginning to the end and a 7-point functional scoring system were used for evaluation. The scoring criteria were as follows: A score of 0 indicated normal traversal with no foot slips; 1 indicated traversal with lateral grasping; 2 reflected walking difficulty but successful completion; 3 indicated slow traversal due to impaired coordination; 4 represented inability to traverse the beam; 5 indicated immobility on the beam; and 6 represented inability to remain on the beam for at least 10 s (Modi et al., 2024; Tung et al., 2016; Urakawa et al., 2007).

#### 2.4.2 Rotarod test

The rotarod test assessed balance, grip strength, and gross motor coordination. The device (Elkimak Engineering and Industrial Design Consultancy, Turkiye) was programmed to accelerate from 0 to 40 revolutions per minute (RPM), with an acceleration step speed of 0.3 RPM, and an unlimited duration (Manno et al., 2023). Before baseline measurements, each animal received training for 20 min per day over three consecutive days. On the test day, a total of eight trials were conducted, with the first three serving as training. After the first three trials, to prevent animal fatigue, 10-min rest period was served before the test sessions. In the Rotarod apparatus, the trigger pressure sensors located beneath each landing platform automatically measured the latency to fall and the RPM. The latency to fall of the average final five trials was evaluated for the test performance (Tung et al., 2014, 2016).

### 2.5 Electrophysiological measurements

Following the behavioral tests, Auditory Brainstem Responses (ABR) and cervical Vestibular Evoked Myogenic Potentials (cVEMP) were consecutively conducted under general anesthesia induced by intraperitoneal injections of ketamine (100 mg/kg) and xylazine (10 mg/kg). Additional doses of anesthetic agents were administered as needed in a controlled manner. Body temperature was maintained between 37 °C and 38 °C throughout the procedure using a heating pad.

#### 2.5.1 Auditory brainstem response (ABR)

The ABRs were recorded to assess auditory function. The tests were conducted at low frequencies (1, 2, 4, and 8 kHz). Subcutaneous needle electrodes were placed with the reference electrode at the midline of the occipital region, the recording electrode behind the stimulated ear, and the ground electrode at the rear leg. Electroencephalographic (EEG) signals were filtered within the range of 100 Hz–3 kHz and averaged over 1024 stimuli at a rate of 25.1 using an insert transducer. Thresholds were initially determined in 10 dB steps, with finer increments of 5 dB near the threshold following the peak II response. Data were presented as ABR thresholds and ABR Peak 1 (P1) amplitudes. Recordings were made using a preamplifier and the SmartEP data acquisition system (Intelligent Hearing Systems, Miami, FL, USA).

#### 2.5.2 Cervical-vestibular evoked myogenic potential (cVEMP)

**FIGURE 2:**
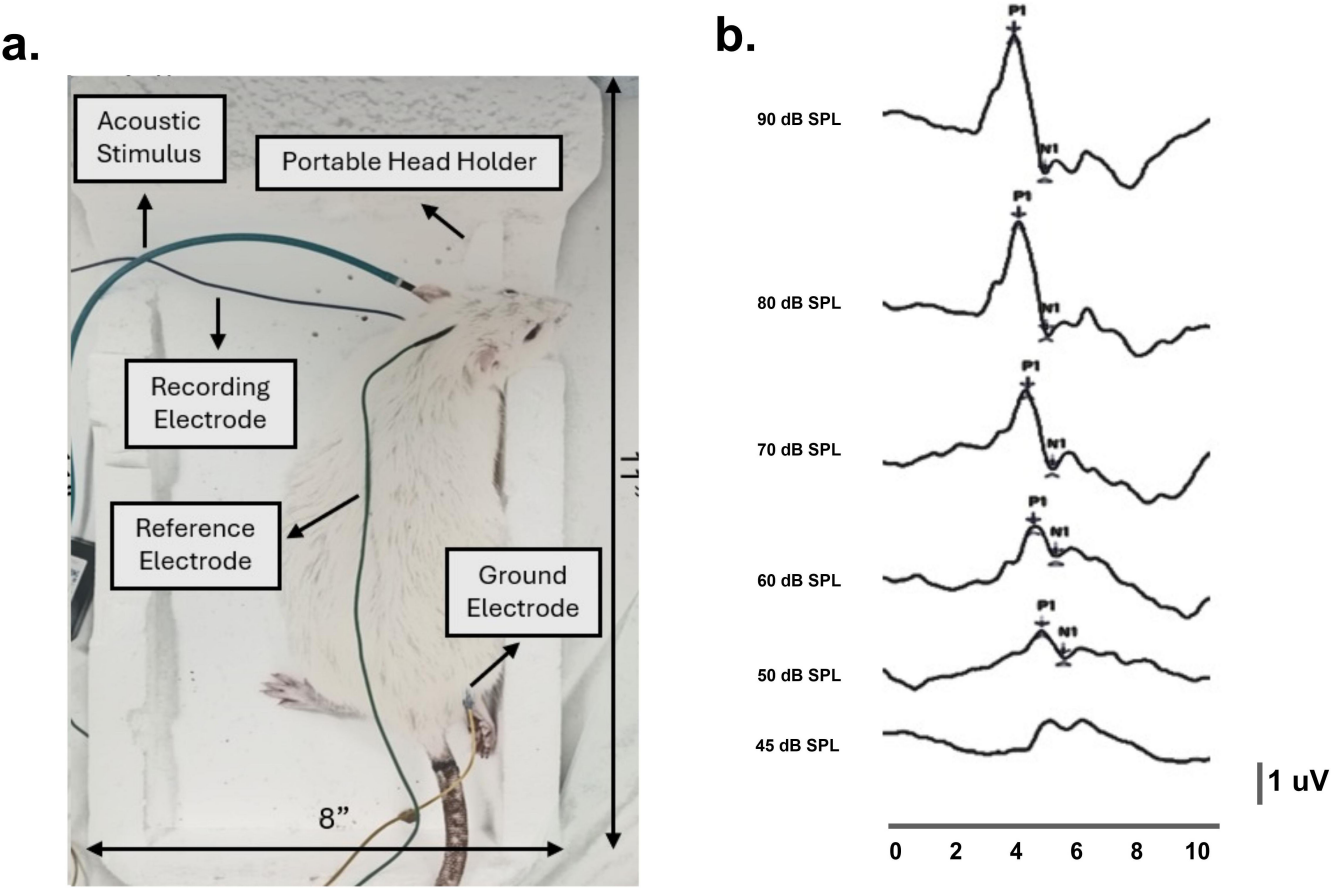
Cervical vestibular myogenic evoked potentials (cVEMP) recording setup and custom-designed sponge holder in a representative rat. **(a)** Using a portable head holder, the rat’s head remained stable at approximately 90° bilateral rotations throughout the procedure. Recording electrodes were placed perpendicularly on the sternocleidomastoid muscles, with reference and ground electrodes positioned at the occipital midline and hind limb. **(b)** In a representative rat, baseline cVEMP responses were recorded across decreasing stimulus intensities (90–45 dB SPL), with corresponding P1–N1 latencies between 3 and 5 ms.

The cVEMP test assessed vestibular functions originating from the otolith organs. The cVEMP responses were recorded from rats’ sternocleidomastoid muscles (SCM) in a modified version of a custom-built holder originally described in Raciti et al. (2023). A portable head holder was used to fix the rat’s head at ∼90° rotation allowing for bilateral potential measurements (Figure 2).

A needle electrode was inserted into the SCM on the tested side, with reference and ground electrodes placed at the occipital midline and hind limb under. Electrode impedance was checked prior to testing, and only recordings with impedance ≤3–5 kΩ were accepted. Electromyographic (EMG) signals were band-pass filtered between 30 and 3000 Hz. 1 kHz tone bursts were delivered monaurally via ER3A insert earphones at intensities ranging from 90 to 40 dB SPL in 10 dB steps. Stimulation was delivered at 5/s rate, with 256 average responses per run. cVEMP responses were deemed normal when a reproducible biphasic waveform (P1–N1) with 3–5 ms latency was observed (Raciti et al., 2023). Data were reported as P1–N1 amplitude, latencies, and thresholds. Recordings were made using the pre-amplifier and the SmartEP data acquisition system (Intelligent Hearing Systems, Miami, FL, USA).

### 2.6 Statistical analysis

All statistical analyses and figures were generated using GraphPad Prism version 10.2.2 (GraphPad Software, RRID:SCR_002798). Normality tests were assessed to determine the use of parametric or non-parametric tests. Two-way ANOVA followed Sidak’s multiple comparisons test between groups was used to evaluate in each test parameter and on the following days. A *p*-value < 0.05 was considered statistically significant.

## 3 Results

**FIGURE 3:**
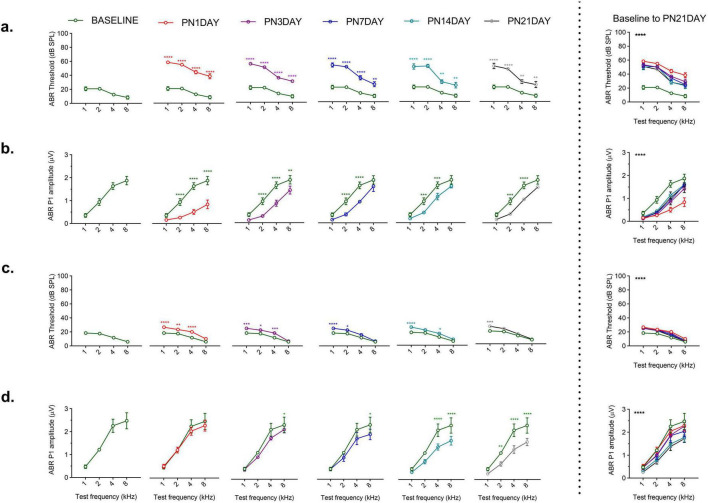
Comparison of ABR P1 threshold and amplitude values following noise exposure. **(a)** In the 120 dB SPL noise exposure group, ABR thresholds dramatically increased across all frequencies on all post-noise days compared to baseline (green curve). **(b)** In the 120 dB SPL noise exposure group, ABR P1 amplitudes were significantly reduced on all post-noise days; the values did not reach baseline over 21 days. **(c)** In the 110 dB SPL noise exposure group, relatively small threshold shifts were observed, with varying degrees of recovery across frequencies over 21 days. **(d)** In the 110 dB SPL noise exposure group, small ABR P1 amplitude shifts were observed on different post-noise days. The graphs to the right of the dashed line show the overall comparisons from baseline to post-noise day 21 across frequencies. ABR P1 amplitudes were measured at 70 dB SPL. Data are presented as mean ± SEM. Statistical analysis: two-way ANOVA with Sidak’s multiple comparisons; ****p < 0.0001, ***p < 0.001, **p < 0.01, *p < 0.05. Asterisks indicate significant differences between baseline and post-noise days at each frequency.

Exposure to 110 dB SPL resulted in small but significant threshold shifts, whereas 120 dB SPL caused sustained and pronounced auditory deficits. Following 110 or 120 dB SPL noise exposure, the auditory function exhibited a distinct time-dependent recovery profile. The 120 dB SPL LFN exposure produced a significant increase in ABR thresholds and a reduction in P1 amplitudes at all post-noise days [thresholds: *F* (15, 75) = 4.179, *p* < 0.0001; amplitudes: *F* (15, 75) = 4.203, *p* < 0.0001] (Figures 3a, b). In contrast, the 110 dB SPL LFN exposure caused small and temporary threshold shifts except at 1 kHz [*F* (3,15) = 115.3, *p* < 0.0001] (Figure 3c). In this group, ABR P1 amplitudes were reduced on different post-noise days at only a few frequencies [*F* (3,15) = 79.11, *p* < 0.0001] (Figure 3d). Interestingly, both groups showed a marked improvement in ABR thresholds and amplitudes within the first 3 days, suggesting a rapid recovery. However, no further significant recovery was obtained from day 3 to day 21, and the values did not fully return to baseline after 120 dB SPL LFN exposure. Notably, the marked improvements in ABR parameters within the first 3 days post-exposure highlight the critical role of the early recovery period.

**FIGURE 4:**
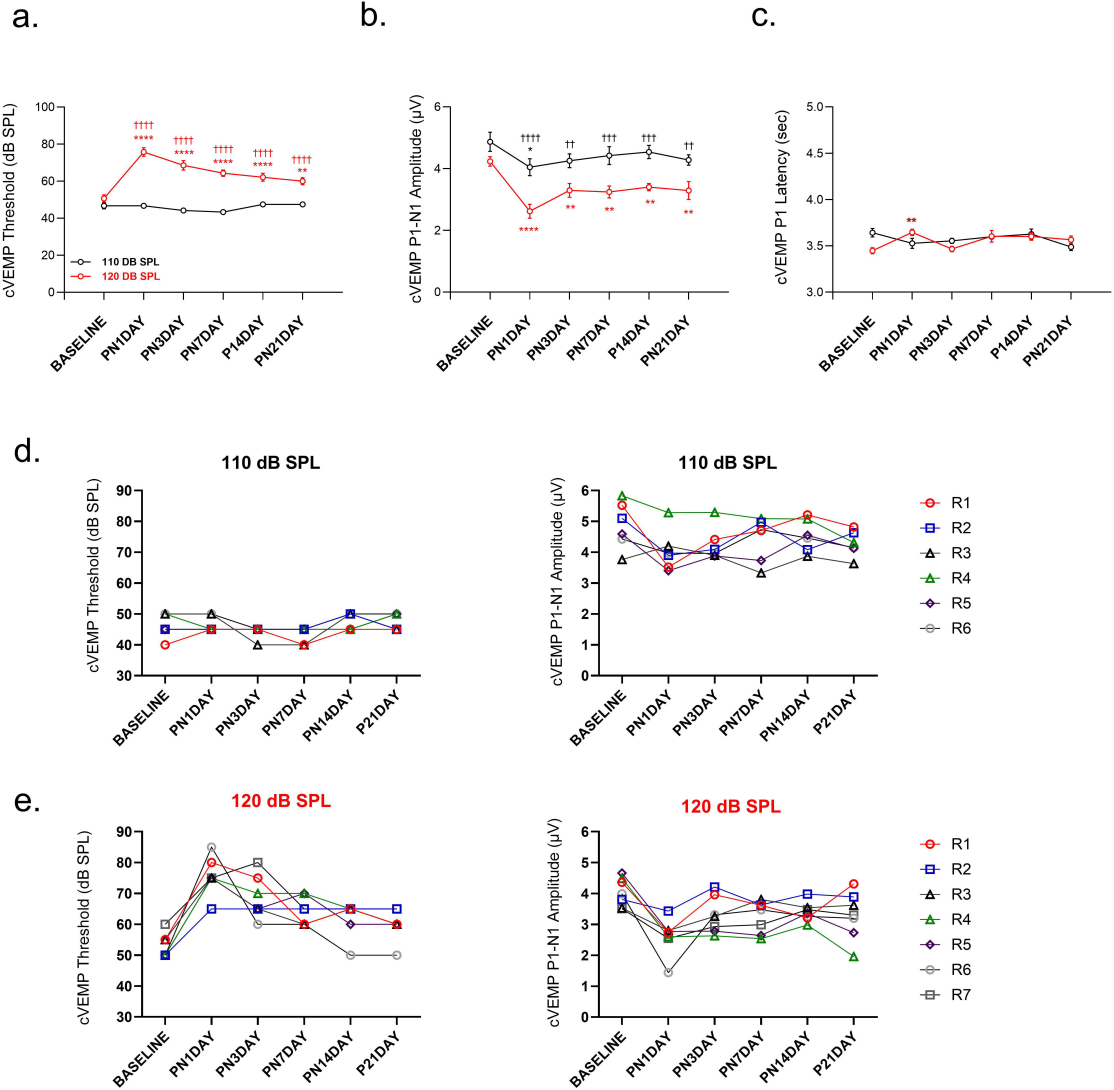
Comparison of vestibular function following noise exposure. **(a)** cVEMP thresholds increased on all post-noise days in the 120 dB SPL group compared to baseline and the 110 dB SPL group, whereas no significant changes were observed in the 110 dB SPL group. **(b)** cVEMP P1–N1 amplitudes were reduced on all post-noise days in the 120 dB SPL group, but only on 1 day in the 110 dB SPL group. **(c)** cVEMP P1 latency was prolonged only on 1 day post-noise in the 120 dB SPL group, but not in the 110 dB SPL group. **(d)** Individual cVEMP analyses showed minimal threshold variability in the 110 dB SPL group, but P1–N1 amplitudes decreased on 1 day post-noise and mostly recovered by day 3. **(e)** In the 120 dB SPL group, individual cVEMP analyses showed that on 21 days, while three animals (42.9%) recovered to their baseline thresholds, their amplitudes remained reduced. Other animals (57.1%) did not reach the baseline values over 21 days, either thresholds or amplitudes. cVEMP amplitudes and latencies were measured at 90 dB SPL. Data are presented as mean ± SEM. Statistical analysis: two-way ANOVA with Sidak’s multiple comparisons; ****p < 0.0001, **p < 0.01, *p < 0.05, ††††*p* < 0.0001, †††*p* < 0.001, ††*p* < 0.01. Asterisks indicate significant differences between baseline and post-noise days; daggers indicate significant differences between 110 dB SPL and 120 dB SPL noise exposure groups. R1–R7 indicate the individual measurements of each rat for each group.

Exposure to 120 dB SPL LFN affects vestibular function permanently. The 120 dB SPL LFN exposure produced a permanent increase in cVEMP thresholds compared to baseline [*F* (5, 55) = 10.64, *p* < 0.0001] (Figure 4a). In contrast, 110 dB SPL LFN exposure did not change the cVEMP thresholds over time. The comparisons between groups revealed significant differences in thresholds on all following days [*F* (1, 11) = 171.8, *p* < 0.0001] (Figure 4a).

The 120 dB SPL LFN exposure produced cVEMP P1-N1 amplitude reduction on all post-noise days compared to baseline [*F* (5, 55) = 10.64, *p* < 0.0001] and compared to the 110 dB SPL [*F* (1, 11) = 19.78, *p* = 0.001]. The average cVEMP P1-N1 amplitude decreased 50% on the 1 day post-noise, and a partial recovery was obtained over subsequent days. However, the values did not reach baseline over the follow-up period [baseline versus PN21DAY t(55) = 4.059; *p* = 0.002] (Figure 4b). In contrast, the 110 dB SPL LFN exposure caused cVEMP P1-N1 amplitude reduction in only 1 day post-noise and recovered in the following days [t(55) = 3.280; *p* = 0.027] (Figure 4b). Furthermore, only in the 120 dB SPL group, cVEMP P1 latency prolonged on the 1 day post-noise compared to baseline [t (55) = 3.674, *p* = 0.008] (Figure 4c).

Individual animal analyses showed that, in the 110 dB SPL group, the cVEMP P1-N1 amplitude decreased on the 1 day post-noise without threshold changes. Moreover, their values recovered on the third day compared to baseline (Figure 4d). In the 120 dB SPL group, all rats showed dramatic cVEMP P1-N1 amplitude reduction on the 1 day post-noise. On 21 days, while three animals (42.9%) recovered to their baseline thresholds, their amplitudes remained reduced (Figure 4e).

**FIGURE 5:**
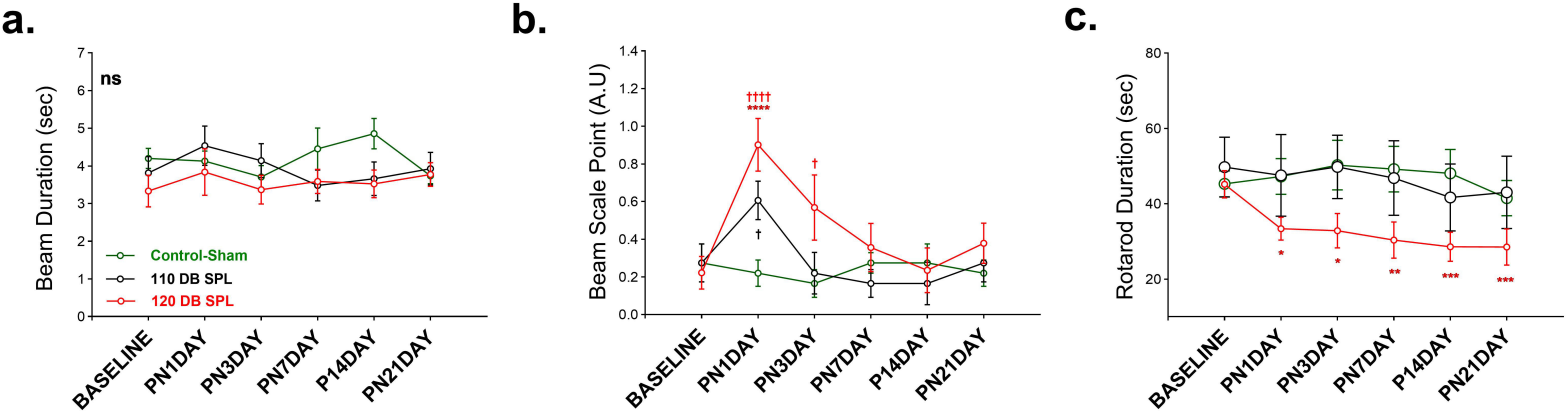
Comparison of behavioral balance performance following noise exposure. Balance performance was assessed using behavioral tests at baseline and multiple post-noise time points. **(a)** Beam Duration Time, which indicates the time spent on the beam for crossing, showed no significant differences across all groups. **(b)** Beam Scale Point presented the function and quality of crossing according to the scale points. The 120 dB SPL noise exposure group showed impaired crossing performance compared to the control-sham group by day 3. The 110 dB SPL noise exposure group performed worse compared to the control-sham group only on 1 day post-noise. **(c)** Rotarod Duration Time, measuring the time spent on the rod until falling. The 120 dB SPL group exhibited a robust and consistent decline across all post-exposure time points, compared to baseline, indicating long-term motor deficits. No significant changes were obtained in the control-sham and 110 dB SPL group over 21 days. Data are presented as mean ± SEM. Statistical analysis: two-way ANOVA with Sidak’s multiple comparisons; ****p < 0.0001, ***p < 0.001, **p < 0.01, *p < 0.05, ††††*p* < 0.0001, †*p* < 0.05. ns, not significant. Asterisks indicate significant differences between baseline and post-noise days; daggers indicate significant differences between control-sham and noise exposure groups. A.U, arbitrary units.

Noise exposure induced balance and motor coordination deficits in an intensity-dependent manner. Balance and motor performance were assessed using the beam walking and rotarod tasks. In the beam duration test, no significant differences were obtained among groups across all time points (Figure 5a). However, beam score analysis revealed a marked effect of noise exposure among groups [*F* (10, 80) = 2.281, *p* = 0.021). The 120 dB SPL LFN exposure caused worse scores compared to the control-sham group on the 1 day post-noise [t (96) = 4.479, *p* < 0.0001] and third day [t(96) = 2.652, *p* = 0.029]. In contrast, the 110 dB SPL group showed worse scores compared to the control-sham group on only 1 day post-noise [t(96) = 2.449, *p* = 0.047] (Figure 5b).

In the rotarod task, the 120 dB SPL LFN exposure led to a lasting impairment at all post-noise days compared to baseline values [*F* (5, 65) = 4.261, *p* = 0.002] (Figure 5c). The 110 dB SPL group showed no difference compared to the control group on any post-noise day. These findings suggest that while 110 dB SPL noise exposure induces only transient motor deficits, 120 dB SPL noise exposure causes long-lasting motor coordination and balance impairments.

## 4 Discussion

This study examines the intensity-dependent long-term effects of noise exposure on the vestibular system in rats, using both electrophysiological and behavioral tests. Numerous studies have described that higher-intensity noise exposure leads to permanent ABR threshold shifts, reduced amplitudes, and incomplete recovery (Berns et al., 2025; Chen et al., 2014; Domarecka et al., 2021; Natarajan et al., 2023). Moreover, both clinical (Akin et al., 2012; Kumar et al., 2010; Snapp et al., 2023; Tseng and Young, 2013) and preclinical researches (Akdogan et al., 2009; Stewart et al., 2016, 2020a,^2021^; Tamura et al., 2012) revealed vestibular system can be sensitive to intense acoustic trauma. Our results show that exposure to 120 dB SPL LFN causes lasting impairment of vestibular and auditory function until 21 days after acoustic trauma. In contrast, 110 dB SPL LFN exposure led to transient effects on both inner ear system functions. This study aligns with the expectation of intensity-dependent damage to the auditory and vestibular system.

Previous studies using VsEPs have reported permanent damage up to 28 days following 120 dB SPL exposure (Stewart et al., 2020a), whereas recovery was observed by Day 7 after 110 dB SPL exposure (Stewart et al., 2021). This may seem at odds with our findings, as in the 110 dB SPL group, we observed measurable damage only on 1 day post-noise. However, this discrepancy likely reflects differences in the sensitivity of the two electrophysiological approaches, i.e., VsEP and cVEMP. VsEPs are evoked by rapid head acceleration (Jones et al., 2011) whereas cVEMPs are elicited by acoustic stimulation (Colebatch and Halmagyi, 1992). Given that the present study aimed to examine noise-induced vestibular dysfunction, using an acoustically evoked test (cVEMP) in rats represents an appropriate model for clinical translation.

Behavioral test results further support the cVEMP findings. Although the 120 dB SPL group showed a permanent decrease in the rotarod performance, the improvements were observed in beam test scores. This suggests that some non-overt deficits may go unnoticed due to vestibular compensation. However, the long-lasting deficits observed during the rotarod test may represent a sign of vestibular dysfunction that becomes apparent under relatively “challenging task” conditions (Luong et al., 2011). This highlights the need to assess subtle impairments using more detailed and demanding tests.

Histological studies in animals have shown that noise exposure can induce vacuolization and nuclear loss in type I hair cells, while type II hair cells, supporting cells, and nerve fibers generally remain intact (Hsu et al., 2008). Furthermore, noise exposure can trigger apoptosis in saccular hair cell bodies (Akdogan et al., 2009), cause stereociliary bundle damage (Stewart et al., 2016), and reduce the number of calyx-only afferent terminals (Stewart et al., 2018). Early reports also suggested that direct mechanical deformation of the otolithic organ walls and disruption of endolymph flow may contribute to these changes (Mangabeira-Albernaz et al., 1959; McCabe and Lawrence, 1958). These findings support the notion that the saccule, due to its anatomical proximity to the stapes footplate and inherent sensitivity to acoustic stimulation, exhibits the earliest and most pronounced functional changes detectable by cVEMP. Because cVEMP primarily reflects saccular function, which is consistent with the literature indicating that the saccule is the most vulnerable vestibular structure to noise exposure (Akdogan et al., 2009; Hsu et al., 2008; Mangabeira-Albernaz et al., 1959; Snapp et al., 2023; Stewart et al., 2016). This vulnerability can result from the high sensitivity of calyceal afferents to mechanical vibrations in the endolymph induced by intense sound (Curthoys and Vulovic, 2011; Fernández and Goldberg, 1976; McCue and Guinan, 1995; Zhu et al., 2011).

In contrast, the semicircular canals are generally less susceptible to noise-induced damage, and any effects tend to be transient or occur only under intense or prolonged exposure. Some studies have reported endolymphatic hydrops or other structural changes in the lateral and posterior semicircular canals following high-intensity noise exposure (Pan et al., 2025), while others have observed minimal or no detectable alterations (McCabe and Lawrence, 1958; Stewart et al., 2016). These observations suggest that although cVEMP provides a sensitive measure of saccular dysfunction, it does not capture the full spectrum of vestibular changes, and semicircular canal involvement may occur under more extreme conditions. In this study, although long-term effects of noise exposure were successfully demonstrated using cVEMP, a more detailed examination of all potential changes in the vestibular end organs may be considered.

Sex hormones have been implicated in modulating auditory and vestibular function. Estrogen has been shown to protect against cochlear injury, whereas androgens may enhance susceptibility (Henry, 2004; Meltser et al., 2008). Estrogen and progesterone receptors have also been identified in vestibular structures (Stenberg et al., 1999, 2001). Female rats may exhibit distinct responses, potentially mediated by estrogen’s protective influence. Nevertheless, available rodent studies provide limited evidence for sex-dependent differences in vestibular vulnerability, and some have reported no significant effect (Pan et al., 2025). In the present study, we employed only male rats to reduce biological variability associated with the estrous cycle; however, this choice may restrict the generalizability of our results. Future studies may consider examining the effects of sex in detail to better understand its role in noise-induced vestibular pathology.

## 5 Conclusion

This study demonstrates that exposure to 120 dB SPL LFN causes long-lasting impairments in vestibular function, whereas 110 dB SPL exposure leads to only transient and less severe effects. In addition, early behavioral recovery may mask underlying subtle deficits through vestibular compensation. These findings also highlight the importance of the long-term effects of vestibular compensation following noise exposure, which may be critical for diagnostic and therapeutic strategies. Even without overt auditory changes, screening for vestibular dysfunction may be essential for early detection and rehabilitation in populations frequently exposed to noise.

## Data Availability

The raw data supporting the conclusions of this article will be made available by the authors, without undue reservation.
